# Factors associated with recent HIV testing among younger gay and bisexual men in New Zealand, 2006-2011

**DOI:** 10.1186/1471-2458-14-294

**Published:** 2014-03-31

**Authors:** Nathan J Lachowsky, Peter JW Saxton, Nigel P Dickson, Anthony J Hughes, Alastair JS Summerlee, Cate E Dewey

**Affiliations:** 1 Department of Population Medicine, University of Guelph, Guelph, Canada; 2 Centre for Public Health and Zoonoses, University of Guelph, Guelph, Canada; 3 AIDS Epidemiology Group, University of Otago, Dunedin, New Zealand; 4 Research Analysis and Information Unit, New Zealand AIDS Foundation, Auckland, New Zealand; 5 Department of Biomedical Science, University of Guelph, Guelph, Canada; 6 University of Guelph, 2509 Stewart Building, OVC, 50 Stone Road East, Guelph, Ontario N1G 2W1, Canada

**Keywords:** Gay men, HIV/AIDS, Surveillance, Testing, Youth, MSM, New Zealand, Sexual behaviour, Health promotion

## Abstract

**Background:**

Understanding HIV testing behaviour is vital to developing evidence-based policy and programming that supports optimal HIV care, support, and prevention. This has not been investigated among younger gay, bisexual, and other men who have sex with men (YMSM, aged 16-29) in New Zealand.

**Methods:**

National HIV sociobehavioural surveillance data from 2006, 2008, and 2011 was pooled to determine the prevalence of recent HIV testing (in the last 12 months) among YMSM. Factors associated with recent testing were determined using manual backward stepwise multivariate logistic regression.

**Results:**

Of 3,352 eligible YMSM, 1,338 (39.9%) reported a recent HIV test. In the final adjusted model, the odds of having a recent HIV test were higher for YMSM who were older, spent more time with other gay men, reported multiple sex partners, had a regular partner for 6-12 months, reported high condom use with casual partners, and disagreed that HIV is a less serious threat nowadays and that an HIV-positive man would disclose before sex. The odds of having a recent HIV test were lower for YMSM who were bisexual, recruited online, reported Pacific Islander or Asian ethnicities, reported no regular partner or one for >3 years, were insertive-only during anal intercourse with a regular partner, and who had less HIV-related knowledge.

**Conclusion:**

A priority for HIV management should be connecting YMSM at risk of infection, but unlikely to test with appropriate testing services. New generations of YMSM require targeted, culturally relevant health promotion that provides accurate understandings about HIV transmission and prevention.

## Background

Access to and timely uptake of HIV testing is a necessary precursor to receiving the most effective care and treatment for people infected with HIV [1]. Processes around testing itself, such as counseling, may even help limit ongoing transmission risks among those uninfected [2-4]. Therefore, effective HIV testing needs to be a vital component of a country’s comprehensive public health policy.

Global increases in HIV prevalence among gay, bisexual and other men who have sex with men (MSM) [5-8], a reflection of both improved survival and ongoing incidence, present major challenges to effective prevention strategies [9,10]. One concern is that MSM with undiagnosed HIV [11], particularly soon after acquisition and consequently not on treatment, account for a disproportionate fraction of new infections; Australian research suggested that 31% of new HIV infections occur from the estimated 9% of MSM with undiagnosed HIV in Australia [12]. In New Zealand, as in many other developed countries, the HIV epidemic continues to predominantly affect MSM [13,14]. A recent community survey in Auckland (New Zealand’s largest city) found that 21% of HIV-infected MSM were undiagnosed [15], suggesting that the proportion of new infections attributable to this group is likely considerable. As well as enabling ongoing spread in the presence of non-condom use, delayed diagnosis means that the most effective treatment cannot be offered to individuals. Using surveillance data, 41% of MSM diagnosed with HIV in New Zealand in the period 2005-2010 presented late (i.e. CD4 count of less than 350 cells per microliter) [16]. These data, and the impact of undiagnosed HIV on transmission, highlight the importance of understanding HIV testing.

Younger MSM (YMSM) – those aged less than 30 years – are no exception: they comprised one quarter (23%) of all new HIV diagnoses among MSM in New Zealand in 2011 [17] and may be at increased risk of undiagnosed infection compared with older MSM [15]. Internationally, HIV epidemiology has shown that YMSM have increasing HIV incidence and are less likely to be aware of their HIV infection [18]. Although the New Zealand AIDS Foundation’s HIV Prevention Plan 2009-2014 identifies the need to focus on younger gay and bisexual men [19], no HIV research in New Zealand has specifically focused on this group. Previous research in New Zealand has examined HIV testing experiences among community samples of MSM [20,21], but no studies have focused on subgroups such as those under the age of 30.

In New Zealand, HIV testing is offered free of charge at sexual health clinics, the New Zealand AIDS Foundation clinics offer rapid testing services, and HIV treatments are publically funded. HIV testing may be measured in a number of ways when used as a research or surveillance parameter. Although “never having tested” is clearly valuable [22-24], the corollary of “ever having been tested” is less useful without an indication of the frequency of testing or the timing of the most recent test [2,25,26]. The most commonly used measure of recent HIV testing is “being tested in the previous 12 months” [7,22,27-31], which we have also chosen in order to facilitate comparisons. The objective of this study was to explore the prevalence of and factors associated with recent HIV testing among YMSM in New Zealand.

## Methods

### Study sites, recruitment and pooled sample

We analyzed data from New Zealand’s HIV sociobehavioural surveillance program for MSM collected in 2006, 2008, and 2011. This consists of two surveys: the Gay Men’s Periodic Sex Survey (GAPSS) and Gay men’s Online Sex Survey (GOSS). Recruitment for GAPSS took place each year over one week in February: the first day at a community fair day and all subsequent days at gay bars and sex-on-site venues. Following GAPSS, the same questionnaire was used for GOSS with participants accessed through Internet dating sites. Eligible participants needed to be at least 16 years old, identify as a man, report having had sex with another man in the past five years, and not have participated in GAPSS or GOSS previously that year. Participants completed a short self-administered anonymous questionnaire. Both surveys received ethical approval from the Northern X Regional Ethics Committee. Detailed descriptions of the methods are described elsewhere [15].

Participant responses from the 2006-2011 rounds of GAPSS and GOSS were pooled. To ensure each individual was represented only once in the pooled sample, a participant’s response was only included if he had not previously participated. Since YMSM who reported testing HIV-positive more than one year prior to the time of survey would not have tested subsequently, they were also excluded. A variable for survey year was included in all analyses to control for any change over time.

A recent review of HIV in YMSM highlighted the variation in and importance of meaningful definitions for “young” or “youth” [18]. We selected an upper age limit of 29 years in order to facilitate comparisons given the U.S. Centers for Disease Control and Prevention definition of young MSM (aged 13-29 years) and the Commonwealth’s definitions of young people (aged 15-29 years).

### Measures

#### ***Dependent variable***

Participants were asked, “Have you ever had an HIV test?” Those who had were asked where (excluding respondents in 2006) and when the last occasion had been. The dependent variable was an HIV test in the previous 12 months.

#### ***Independent variables***

Participants were asked a number of questions about their knowledge, attitudes and behaviour related to HIV. Questions on sexual behaviour in the previous six months included specifically asking if they had had sex with any man or any woman. An ordinal scale was used to enumerate male sexual partners. Respondents were asked separately about regular sex partners, being men they had sex with four or more times, and casual sex partners, being men they had sex with on fewer occasions during that period. Those with a current regular partner were asked the duration of the relationship, and whether he was a ‘boyfriend’-type (including boyfriend, long-term lover, life partner, or civil union partner) or ‘fuckbuddy’-type partner (including fuckbuddy or “friend I have sex with”). Those who had engaged in anal intercourse were asked separately about condom use with their regular and/or casual partners, for both insertive and receptive anal intercourse. Data on condom use were collected on a five-level scale that was collapsed into three-levels: low (“very rarely” or “never” used), medium (“about half the time”), and high (“always” or “almost always”).

Various attitudinal statements related to condom use (e.g. “Condoms are OK as part of sex”) and HIV transmission (e.g. “A man who knows he has HIV would tell me he was positive before we had sex”) were presented and the participants were asked to state their agreement with these on a four-point scale dichotomized into “disagree” or “agree”. A single measure of HIV-related knowledge was assessed by responses to the statement, “HIV cannot pass through an undamaged latex condom”.

Socio-demographic information included age (in years), sexual identity, ethnicity, level of education, and attachment to the gay community (i.e. amount of time spent with other gay men, dichotomized as “a lot” or “some” versus “little” or “none”). Sexual identity responses included gay (or homosexual), bisexual, or “other”. Multiple ethnicity responses were permitted and grouped into New Zealand European/Pākehā, Māori, Pacific, Asian, and “other” using a commonly used standard prioritization system [32]. Education level was dichotomized into less than tertiary/post-secondary education and at least some tertiary/post-secondary education.

Respondents were asked if they had been for a sexual health check-up or treatment in the past year for any sexually transmitted infection (STI), and then if they had been diagnosed with one or more of a list of STIs in that period. Respondents were also asked what they believed their own HIV status was at the time of survey: “definitely negative”, “probably negative”, “don’t know”, or “definitely” or “probably positive”.

### Data analysis

Data were analyzed to determine the prevalence of recent HIV testing and associated factors. Data analyses were conducted using the statistical package StataSE 11.2 [33] and a p-value of less than 0.05 was considered significant unless otherwise specified.

It was hypothesized that recent HIV testing would be associated with three potential collinear variables (i.e. increased odds of recent HIV testing would be positively associated with recent sexual health testing/treatment, recent STI diagnosis, and greater certainty of “belief of one’s own HIV status”), evaluated using simple logistic regression. These variables were excluded from the multivariate analyses (see below).

Independent factors associated with recent HIV testing were determined using logistic regression. Univariate analyses were conducted to screen independent variables for an association with the outcome variable using a liberal p-value of 0.2. The final multivariate model was built using a manual backward-elimination approach. Likelihood ratio tests were used to test overall significance of variables prior to removal. Collinear variables were excluded from the analyses (i.e. the variable for number of regular partners in the past six months was excluded in lieu of the total number of sexual partners in the past six months). All variables with a significant p-value of <0.05 were retained. Confounding was assessed throughout model development; if adding or removing a variable resulted in greater than a 20-30% change of an independent variable’s coefficient it was retained in the model [34].

## Results

There were a total of 9,595 responses across the 2006, 2008, and 2011 rounds of GAPSS and GOSS surveillance. Of these, 3352 were men aged between 16 and 29 years who were included in the analysis (1341 responses from 2006, 881 responses from 2008, and 1130 responses from 2011), having removed 354 men who had answered a survey in a previous round, 31 who did not complete the question on HIV testing, and 4 who had tested HIV-positive more than one year prior to the survey. The mean age was 23.2 years (SD = 3.5) with 17.6% aged 16-19, 44.8% aged 20-24, and 37.7% aged 25-29.

Overall, 39.9% (n = 1338/3352) of YMSM reported an HIV test in the previous year (compared with 40.0% of MSM older than 29). Twenty-four YMSM (0.7% of total sample) reported an HIV-positive test result in the past year (20 of these in the past 6 months). The prevalence of and univariate associations with recent HIV testing for each category of independent variable are presented in Table 1. Of YMSM who had tested recently (data collected in 2008 and 2011, n = 840), 47.5% tested with their general practitioner or doctor, 38.7% at a sexual health clinic, 10% at a New Zealand AIDS Foundation clinic, and the remaining 3.8% elsewhere unspecified.

**Table 1:** Factors associated with having an HIV test within 12 months of the survey date among YMSM in New Zealand 2006, 2008, 2011 based on univariate analyses

	**n**	**Recent test %**	**Univariate OR (95% CI)**	**p**
**Demographics**				
Age (16-29 years old)				
OR for each per year increase in age	-	-	**1.06 (1.03-1.08)**	***
Recruitment site				
Fair day	896	46.8	1.00 (ref.)	
Gay bars	120	49.2	1.15 (0.78 – 1.68)	
Sex-on-site venues	76	54.0	1.40 (0.88 – 2.25)	
Internet dating sites	2260	36.2	**0.65 (0.56 -0.76)**	***
Identity				
Gay	2345	44.3	1.00 (ref.)	
Bisexual	804	28.9	**0.51 (0.43 – 0.60)**	***
Other	192	33.9	**0.66 (0.48 – 0.90)**	**
Ethnicity				
European/Pākehā^a^	2277	40.7	1.00 (ref.)	
Māori	398	39.2	0.92 (0.74 – 1.15)	
Pacific	144	30.6	**0.63 (0.44 – 0.91)**	*
Asian	307	38.4	0.87 (0.68 – 1.12)	
Other	197	44.7	1.13 (0.84 – 1.52)	
Education				
Less than tertiary	2180	37.3	1.00 (ref.)	
At least some tertiary	1140	45.0	**1.37 (1.19 – 1.59)**	***
Time spent with other gay men				
Little or none	1443	31.3	1.00 (ref.)	
Some or a lot	1858	46.7	**1.97 (1.70 – 2.28)**	***
**Sexual behavior in last six months**				
Sex with woman				
Yes	592	31.9	**0.66 (0.55 – 0.80)**	***
No	2743	41.6	1.00 (ref.)	
Sex with a man				
Yes	3015	42.7	**4.20 (3.08 – 5.71)**	***
No	333	15.3	1.00 (ref.)	
Number of male sex partners				
0	319	15.1	**0.36 (0.25 – 0.50)**	***
1	670	32.7	1.00 (ref.)	
2-5	1362	41.4	**1.46 (1.20 – 1.78)**	***
6-10	443	45.8	**1.75 (1.36 – 2.24)**	***
11-20	260	60.4	**3.23 (2.40 – 4.35)**	***
>20	241	51.5	**2.24 (1.66 – 3.03)**	***
Number of regular partners				
0	618	36.3	**0.78 (0.64 – 0.96)**	*
1	1288	41.9	1.00 (Ref.)	
2	557	47.4	**1.25 (1.02 – 1.53)**	*
3-4	327	47.7	1.26 (0.99 – 1.61)	
5+	189	46.0	1.17 (0.93 – 1.59)	
Regular partner, current status				
Yes, have a current regular partner	1402	47.7	**1.75 (1.52 – 2.02)**	***
No, do not have a current regular partner	1888	34.4	1.00 (ref.)	
Current regular partner, type				
Boyfriend-type partner	801	48.4	1.00 (ref.)	
Fuckbuddy-type partner	568	46.5	0.91 (0.74 – 1.13)	
Current regular partner, relationship length				
<6 months	558	48.8	1.00 (ref.)	
6 months – 1 year	256	57.4	**1.43 (1.06 – 1.93)**	*
>1 – 2 years	300	46.3	0.90 (0.68 – 1.20)	
3 or more years	266	38.0	**0.65 (0.48 – 0.87)**	**
Current regular partner, anal modality				
Receptive only	276	47.5	0.79 (0.60 – 1.04)	
Insertive only	223	43.1	**0.67 (0.50 – 0.91)**	*
Both insertive and receptive	745	52.4	1.00 (ref.)	
Current regular partner, condom use				
Have a current regular partner, but no anal intercourse	142	34.5	**0.59 (0.40 – 0.87)**	*
Low^b^ condom use	502	47.4	1.00 (ref.)	
Medium^c^ condom use	155	57.4	**1.49 (1.04 – 2.15)**	*
High^d^ condom use	573	49.4	1.07 (0.84 – 1.36)	
Casual partner, any sex				
Yes	2342	44.7	**1.96 (1.67 – 2.32)**	***
No	917	28.9	1.00 (ref.)	
Casual partner, anal modality				
Receptive only	404	43.6	**0.76 (0.60 – 0.96)**	*
Insertive only	426	42.4	**0.73 (0.58 – 0.91)**	**
Both insertive and receptive	1040	50.4	1.00 (ref.)	
Casual partner, condom use				
Casual partners, but no anal intercourse	454	33.9	0.95 (0.68 – 1.33)	
Low^b^ condom use	227	35.2	1.00 (ref.)	
Medium^c^ condom use	269	42.8	1.34 (0.93 – 1.94)	
High^d^ condom use	1386	50.1	**1.82 (1.36 – 2.44)**	***
**Knowledge and attitudes**				
“HIV cannot pass through anundamaged latex condom”				
Knew that	2442	42.1	**1.44 (1.23 – 1.69)**	***
Wasn’t sure/didn’t know	894	33.9	1.00 (ref.)	
“HIV/AIDS is a less serious threat than it used to be because of new treatments”				
Agree	673	36.4	1.00 (ref.)	
Disagree	2659	40.8	**1.26 (1.05 – 1.50)**	*
“A man who knows he has HIV would tell me he was positive before we had sex”				
Agree	1500	35.5	1.00 (ref.)	
Disagree	1819	43.8	**1.43 (1.25 – 1.65)**	***
“I would sometimes rather risk HIV transmission than use a condom during anal sex”				
Agree	445	39.6	1.00 (ref.)	
Disagree	2869	40.2	1.04 (0.85 – 1.27)	
“Condoms are OK as part of sex”				
Agree	3159	40.4	1.00 (ref.)	
Disagree	173	32.4	0.72 (0.52 – 1.00)	
“I don’t like wearing condoms because they reduce sensitivity”				
Agree	1219	40.0	1.00 (ref.)	
Disagree	2086	40.0	1.01 (0.87 – 1.16)	

*OR* adjusted odds ratio with 95%, *CI* confidence interval, *ref.* reference group. Table omits missing data. Bolded text indicates statistical significance at p<0.05. Rows indicating “no current regular partner” or “no casual partners” are excluded from those variables where additional information is provided (e.g. regular partner type, relationship length, anal modality, condom use).

*p < 0.05, **p < 0.01, ***p < 0.001.

^a^Pākehā = indigenous Māori term for European settlers/ethnicity; ^b^“low” condom use refers to very rarely or never using condoms; ^c^“medium” condom use refers to using condoms about half the time; ^d^“high” condom use refers to always or almost always using condoms.

Descriptive statistics and measures of association for the three hypothesized collinear variables with recent HIV testing are shown in Table 2. YMSM who had sexual health testing or treatment in the past 12 months were more likely to have also had a recent HIV test (70.8%) compared with those who had not (12.5%, p < 0.001). Those with a recent STI diagnosis were also more likely to report recent HIV testing (61.6%) compared with YMSM without a recent STI diagnosis (38.0%, p < 0.001). Recent HIV testing among YMSM who believed their own HIV status at the time of survey was “definitely negative” (44.7%) was more likely than among YMSM who believed they were “probably negative” or “didn’t know” (35.3% and 12.8% respectively, p < 0.001) and those who believed they were “probably positive” (17.9%, p < 0.05).

**Table 2:** Descriptive statistics and univariate analyses of potential collinear variables with recent HIV testing of younger MSM in New Zealand 2006, 2008, 2011

	**n**	**Recent test %**	**Univariate OR (95% CI)**	**p**
Sexual health checkup or treatment^a^				
Yes	1552	70.8	**17.76 (14.81 – 21.31)**	***
No	1755	12.5	1.00 (ref.)	
Recent STI diagnosis^a^				
Yes	294	61.6	**2.70 (2.11 – 3.46)**	***
No	2975	38.0	1.00 (ref.)	
Belief of own HIV status				
Definitely positive	29	75.9	**3.85 (1.63 – 9.07)**	**
Probably positive	28	17.9	**0.28 (0.11 – 0.74)**	*
Probably negative	921	35.3	**0.67 (0.57 – 0.79)**	***
Definitely negative	2132	44.7	1.00 (ref.)	
Don’t know	218	12.8	**0.18 (0.12 – 0.28)**	***

*OR* adjusted odds ratio with 95%; *CI* confidence interval, *ref.* reference group. *STI* sexually transmitted infection. Table omits missing data. Bolded text indicates statistical significance at p<0.05.

*p < 0.05, **p < 0.01, ***p < 0.001.

^a^in the last 12 months.

The multivariate model (see Table 3) contains twelve significant factors: five socio-demographic variables, four sexual behaviour variables, two measures of attitude and one of knowledge. The odds of having a recent HIV test were higher among certain YMSM: older YMSM [each per year increase in age (adjusted odds ratio [AOR], 1.06; 95% confidence intervals [95% CI], 1.03-1.08); men who spent at least some time with other gay men compared with little or no time (AOR, 1.44; 95% CI, 1.20-1.73); YMSM reporting multiple sex partners versus one in the last 6 months (e.g. for 2-5 partners AOR, 1.73; 95% CI, 1.32-2.27); YMSM who reported always or almost always using condoms with casual partners compared with very rarely or never using condoms (AOR, 1.51; 95% CI, 1.08-2.10); YMSM who knew “HIV cannot pass through an undamaged latex condom” compared with those who didn’t know or were unsure (AOR, 1.35; 95% CI, 1.12-1.62); those who disagreed with the statement that “HIV/AIDS is a less serious threat than it used to be because of new treatments” (AOR, 1.27; 95% CI, 1.03-1.57); and those who disagreed that “a man who knows he has HIV would tell me he was positive before we had sex” (AOR, 1.28; 95% CI, 1.09-1.51).

**Table 3:** Factors associated with having an HIV test within 12 months of the survey date among YMSM in New Zealand 2006, 2008, 2011 based on multivariate analyses (n = 2908)

	**AOR (95% CI)**	
**Demographics**		
Age (16-29 years old) (AOR for each per year increase in age)	**1.06 (1.03 – 1.08)**	***
Recruitment site (ref: Fair day)		
Gay bars	1.04 (0.66 – 1.64)	
Sex-on-site venue	1.70 (0.94 – 3.08)	
Internet dating sites	**0.73 (0.60 – 0.89)**	**
Identity (ref: gay)		
Bisexual	**0.70 (0.57 – 0.86)**	**
Other	0.86 (0.59 – 1.24)	
Ethnicity (ref: European/Pākehā^a^)		
Māori	0.88 (0.68 -1.15)	
Pacific Islander	**0.61 (0.40 – 0.92)**	*
Asian	**0.71 (0.54 – 0.94)**	*
Other	1.15 (0.81 – 1.62)	
Time spent with other gay men (ref: a little or none)		
Some time or a lot of time	**1.44 (1.20 – 1.73)**	***
**Sexual behavior in the last 6 months**		
Number of men sex partners (ref: one partner)		
None	0.67 (0.45 – 1.01)	
2-5	**1.73 (1.32 – 2.27)**	***
6-10	**1.82 (1.31 – 2.55)**	***
11-20	**3.13 (2.14 – 4.60)**	***
>20	**2.30 (1.56 – 3.40)**	***
Current regular partner, relationship length (ref: <6 months)		
No current regular partner	**0.55 (0.42 – 0.71)**	***
6 months – 1 year	**1.69 (1.19 – 2.41)**	**
>1 – 2 years	0.88 (0.63 – 1.22)	
3 or more years	**0.50 (0.35 – 0.71)**	***
Current regular partner, anal modality (ref: both receptive and insertive)		
Receptive only	0.76 (0.56 – 1.03)	
Insertive only	**0.61 (0.44 – 0.85)**	**
No current regular partner	**0.55 (0.42 – 0.71)**	***
Casual partner, condom use (ref: low condom use^b^)		
No casual partners	1.03 (0.69 – 1.53)	
Casual partners, but no anal intercourse	1.05 (0.71 – 1.55)	
Medium condom use^c^	1.16 (0.78 – 1.74)	
High condom use^d^	**1.51 (1.08 – 2.10)**	*
**Knowledge and attitudes**		
Knew “HIV cannot pass through an undamaged latex condom” (ref: did not know or was unsure)	**1.35 (1.12 – 1.62)**	**
Disagreed “HIV/AIDS is a less serious threat than it used to be because of new treatments” (ref: agreed)	**1.27 (1.03 – 1.57)**	*
Disagreed “A man who knows he has HIV would tell me he was positive before we had sex” (ref: agreed)	**1.28 (1.09 – 1.51)**	**

*AOR* adjusted odds ratio with 95%; *CI*, confidence interval; *Ref* reference group. Observations with missing data on any variable excluded. Bolded text indicates statistical significance at p<0.05.

*p < 0.05, **p < 0.01, ***p < 0.001.

^a^Pākehā = indigenous Māori term for European settlers/ethnicity. ^b^“low” condom use refers to very rarely or never using condoms; ^c^“medium” condom use refers to using condoms about half the time; ^d^“high” condom use refers to always or almost always using condoms.

After controlling for other variables in the multivariate model, the odds of having a recent HIV test were lower among YMSM: who were recruited from Internet dating sites compared with those from the community fair day (AOR, 0.73; 95% CI, 0.60-0.89); who reported a bisexual identity compared with a gay identity (AOR, 0.70; 95% CI, 0.57-0.86); self-identified with Pacific (AOR, 0.61; 95% CI, 0.40-0.92) or Asian (AOR, 0.71; 95% CI, 0.54-0.94) ethnicities compared with European/Pākehā ethnicities; and who were insertive-only during anal intercourse with a regular partner compared with being both insertive and receptive (AOR, 0.61; 95% CI, 0.44-0.85).

A final variable related to regular partner relationship length was significant in the multivariate model. YMSM who reported no current regular partner (AOR, 0.55; 95% CI, 0.42-0.71)] were less likely to have had a recent HIV test compared with YMSM who had been with their current regular partner for less than six months. However, compared with YMSM who had been in a relationship with their current regular partner for less than six months, the odds of recent HIV testing was higher among YMSM in a 6-12 month relationship (AOR, 1.69; 95% CI, 1.19-2.41), but lower among YMSM in relationships longer than three years (AOR, 0.50; 95% CI, 0.35-0.71).

## Discussion

Using the 2006-2011 rounds of sociobehavioural HIV surveillance data from New Zealand, nearly 40% (39.9%, n = 1338/3352) of younger gay, bisexual, and other men who have sex with men (YMSM, aged 16-29) reported testing for HIV in the previous 12 months. The prevalence of recent HIV testing among YMSM in our study was lower than that reported by YMSM in the United States (67%) [27] or Australia (78.8%) [22] although these studies did not include online recruited YMSM (who were less likely to have tested in this sample). Among MSM of all ages from Australia, France, Spain, the United Kingdom and the United States, the prevalence of recent HIV testing ranged between 25-75% in the early 2000s [7]. While comparing testing rates across countries may provide some benchmark, establishing what the appropriate level of recent testing should be is somewhat arbitrary. Actual HIV prevalence among MSM in New Zealand is lower compared with many other Western industrial countries [15], which may affect notions of risk and the need to test.

Factors associated with recent HIV testing among YMSM in New Zealand could be attributed to one of two main themes (see Table 4): HIV risk and health promotion. “HIV risk” factors such as number of sexual partners, length of relationship, anal intercourse modality with a current regular partner, and condom use with casual partners were associated with recent HIV testing behaviour. “Health promotion” factors associated with recent HIV resting included age, sexual identity, ethnicity, time spent with other gay men, as well as HIV-related knowledge and attitudes.

**Table 4:** Factors associated with decreased odds (p < 0.05) of having an HIV test within 12 months of the survey date among YMSM in New Zealand 2006, 2008, 2011

**“HIV risk” factors that decreased odds of recent HIV testing (<12 months)**	**“Health promotion” factors that decreased odds of recent HIV testing (<12 months)**
• Report only one male sex partner in last 6 months	• Younger age
• Report no current regular partner, or a regular partner of longer than 3 years	• Recruited from Internet dating sites
• Having insertive-only anal intercourse with regular partner in last 6 months	• Self-identify as bisexual
• Report low condom use (very rarely or never) during anal intercourse with casual partners in last 6 months	• Self-identify with Pacific or Asian ethnicities
	• Spend a little or no time with other gay men
	• Not know or be unsure that “HIV cannot pass through an undamaged latex condom”
	• Agree “HIV/AIDS is a less serious threat than it used to be because of new treatments”
	• Agree “a man who knows he has HIV would tell me he was positive before we had sex”

### HIV risk

Research from the United States has estimated that the majority (68%) of new HIV infections among MSM occur from their main sexual partner [35]. Among YMSM with a current regular partner in our study, recent testing did not vary by type of regular partner (i.e. “fuckbuddy” or “boyfriend”), but did by anal modality and length of relationship. YMSM who reported being insertive-only during anal intercourse with their regular partner were less likely to have reported a recent HIV test compared with those that were both insertive and receptive. More research is needed to investigate behavioural differences among men who are either exclusively insertive or receptive and men who are both. While previous research has identified differences by anal modalities for condom use and STI testing [36,37], this is one of the first studies to demonstrate the association with recent HIV testing among YMSM [38]. Previous research has identified that insertive-only MSM may perceive their lower risk for HIV infection compared with receptive partners as sufficient reason not to test [39]; this rationale also contextualizes increases in unprotected insertive anal intercourse with an HIV-positive partner among MSM in England from 2001 to 2008 [40]. Relationship duration is important to YMSM’s recent HIV testing behaviour and the associations between testing over time and extra-relational sex needs further exploration.

Some respondents reported numerous behaviours associated with increased risk of HIV transmission, such as low condom use and high numbers of sexual partners (e.g. 7.3% of YMSM reported 20 or more sexual partners in the last six months [n = 241/3295], and 12.1% of YMSM who had anal intercourse with casual partners reported low condom use [n = 227/1882]). Although our research identified lower odds of testing among some YMSM at increased risk of infection, in contrast, there was also evidence of good sexual health practices with young men taking additional precaution. For example, YMSM who reported more sexual partners were more likely to have tested recently, a pattern identified in previous research [2,3,22,28]. Further, YMSM in our study who used condoms more frequently during anal intercourse with casual partners were also more likely test recently [28]. However, condom use in regular relationships was not an associated factor. Our findings, which separate condom use by partner type, may help contextualize previous research findings of both positive and null associations between HIV testing and “any unprotected anal intercourse” [22,24,37].

### Health promotion

A number of YMSM sub-populations were identified who may be unreached or underserviced in HIV testing services. In our study, YMSM who self-reported Pacific and Asian ethnicities were less likely to report recent HIV testing when compared with YMSM with European ethnicities. Consistent with earlier New Zealand research on ever testing for HIV among MSM [21], there were no differences found between recent HIV testing among European and indigenous Māori YMSM. The prevalence of and disparities between HIV testing among different ethnicities vary by country [2,3,22,24,29,31,41-43]. This may be due to different structural contexts and histories of colonization and racialization, and warrants more purposive investigation. Research is needed to understand testing service accessibility, such as that recently done in the United Kingdom, which explored and found no difference in the offer or uptake of HIV testing services between MSM of different ethnicities at sexual health clinics [44].

Education is a common protective factor for young men [2], but while associated with increased testing in many other studies [22,24,27,31,45] it was no longer associated with recent HIV testing after controlling for other factors in our study. However, the knowledge measure (condom efficacy to prevent HIV transmission) and two of the five attitude measures (current perceptions of the threat of HIV and likelihood of HIV-positive disclosure to sexual partners) were associated with recent HIV testing. The current findings contribute to other attitudinal research (e.g. “treatment optimism”) [46], which underscores the continued importance of considering knowledge and attitudes in health promotion.

There was a positive effect of ageing on YMSM’s recent HIV testing behaviour, which is supported by previous research [2,24,45]. YMSM recruited online were less likely to report a recent HIV test, as was found in recent Australian research among MSM of all ages [28]. YMSM in the current study who reported spending more time with other gay men were more likely to report a recent HIV test, which concurs with previous research measuring community-attachment and/or social support [25,28,45]. In the final multivariate model, bisexual-identified YMSM were still less likely to have tested recently compared with gay-identified YMSM. This association no longer existed in a multivariate analysis of Australian MSM of all ages [28], but is consistent with other international work [23,24,27]. More purposive exploration of HIV testing among YMSM with either bisexual identity or behavior is warranted [47]. Taken collectively, lower recent HIV testing prevalence among younger YMSM recruited online, who identify as bisexual, self-report Pacific and Asian ethnicities, and spend less time with other gay men signal opportunities for more targeted health promotion for YMSM in New Zealand.

#### ***Strengths and weaknesses of current study***

This study is based on data from a large and diverse sample collected through a well established HIV behavioural surveillance system reflecting World Health Organization guidelines [48]. As with virtually all studies on MSM, the absence of census data on sexual orientation means that the representativeness of this sample cannot be traditionally assessed [49]. In 2008, the New Zealand resident male population aged 15-59 was 1,290,600; assuming a 2% prevalence of same-sex behaviour this enumerates 25,812 MSM [14], approximately 45% of whom live in Auckland [49]. We believe our sample represents a broad cross-section of YMSM who participate in both social and sexual community settings in New Zealand. The focus on recent HIV testing in this paper provides more temporally relevant evidence to inform and help evaluate health promotion and public health policy, and should be used to contextualize passive HIV surveillance data.

Limitations of the current study include the possibility of reverse causal inferences among the associations found (e.g. decreased condom use as a result of HIV-negative testing experiences versus getting tested as a result of low condom use) given the repeated cross-sectional study design. While increasing age was associated with increased odds of recent HIV testing in this research, it cannot be distinguished from these data whether this was a cohort effect or ageing per se. The use of a pooled sample, necessitating the removal of duplicate and more recent surveys from a given individual, resulted in a slightly younger and earlier sampled group of YMSM. Prior to removing individuals’ multiple observations from the pooled sample the proportion of YMSM who reported a recent HIV test was numerically higher (41.3%) compared with our final sample (39.9%). Use of a pooled sample allowed for these analyses to be conducted on this sub-population of YMSM, but only presents a composite picture of factors associated with recent HIV testing within this time frame; a trend analysis would also be interesting and help inform and potential contextualize health promotion programming and evaluations. Structural factors are important to behavioural understandings [50], but were not included within the questionnaire due to time constraints (e.g. proximity to HIV testing clinics or services, young men’s mobility and migration, or sexual health education).

#### ***Implications and future research***

Given that YMSM with HIV in New Zealand may be more likely than older MSM to be unaware of their infection [15], increasing recent HIV testing for YMSM with potential exposure is particularly important. Further, attitudinal factors associated with recent HIV testing highlight the importance of health promotion work to shape sexual health norms, particularly among younger and new generations of gay, bisexual, and other MSM. Internationally, self-perceptions of risk only partially explain MSM’s decisions to test [3,27]; men reported that their personal responsibility, responsibility towards their partner, and uncertainty of their HIV status were also important motives [51]. Other research has challenged YMSM’s self-perceptions of being at low risk for HIV as reasons for not testing (e.g. when participants reported unprotected anal intercourse and multiple sexual partners but also felt at “low-risk”) [24,31,52].

Not all testing services are equally accessible or preferred [2,52,53] and future research should investigate barriers that some YMSM may face in accessing testing information and services. Lower odds of testing in our study among younger YMSM, bisexual identified YMSM, and YMSM of Pacific and Asian ethnicities are also of concern, as these communities may already experience disenfranchisement from healthcare systems or may not respond as well to general social marketing campaigns [24,28,45,52]. Our findings support recommendations to develop specific and culturally relevant health promotion for these sub-groups [2] and to evaluate their success [54,55].

While the New Zealand Ministry of Health has current recommendations for opt-in HIV testing given verbal informed consent by adults with an identified risk of infection [56], a report on New Zealand’s HIV testing policy is currently in development. In Australia, testing guidelines encourage certain MSM to test every 3-6 months, but that only occurred for 61.1% of those MSM [22]. The responsibility for achieving testing guideline outcomes is a shared responsibility of the individual, physician, public health groups, and government. Given that 47.5% of YMSM in New Zealand had their most recent test with their general practitioner/doctor, research, policy, and programming must also address physicians’ capacity and practice of encouraging testing [57,58].

## Conclusions

New Zealand has maintained a strong health promotion focus on consistent condom use for MSM, but HIV testing is also important to HIV prevention and care. As 40% of younger MSM report recently testing for HIV, there is opportunity for improved uptake of testing services. Testing targets should reflect the realities of local epidemics and should focus on developing appropriate cultures of testing which are commensurate with an individual’s risk, especially for MSM who are at risk of infection but less likely to be tested. New generations of YMSM require sexual health education and targeted health promotion that considers their needs and provides accurate understandings about HIV transmission and prevention.

## Competing interests

The authors declare they have no competing interests.

## Authors’ contributions

NL conceived of the research question, performed the statistical analysis, and drafted the manuscript. PS, ND, and AH designed and coordinated the Gay Auckland Periodic Sex Surveys and Gay men’s Online Sex Surveys. AS and CD helped to draft the manuscript and support statistical analyses. All authors read and approved the final manuscript.

## Authors’ information

NL (BSc) is a doctoral candidate of epidemiology in the Department of Population Medicine at the University of Guelph. PS (PhD) is Director of the Gay Men’s Sexual Health research group in the Department of Social and Community Health at the University of Auckland. ND (MBBS DipEpid (London) MRCP (UK) FRACP FAFPHM) is the Director of the AIDS Epidemiology Group and Associate Professor in the Department of Preventive and Social Medicine in the Dunedin School of Medicine at the University of Otago. AH (MSc) is the Scientific Director in the Research Analysis and Information Unit at the New Zealand AIDS Foundation. AS (PhD) is a Professor of Biomedical Science and President at the University of Guelph. CD (PhD) is a Professor and the Chair of the Department of Population Medicine at the University of Guelph.

## Pre-publication history

The pre-publication history for this paper can be accessed here:

http://www.biomedcentral.com/1471-2458/14/294/prepub
